# Mitochondrial metabolic genes provide phylogeographic relationships of global collections of *Aedes aegypti* (Diptera: Culicidae)

**DOI:** 10.1371/journal.pone.0235430

**Published:** 2020-07-28

**Authors:** H. S. D. Fernando, Menaka Hapugoda, Rushika Perera, William C. Black IV, B. G. D. N. K. De Silva

**Affiliations:** 1 Department of Zoology, Center for Biotechnology, University of Sri Jayewardenepura, Nugegoda, Sri Lanka; 2 Molecular Medicine Unit, Faculty of Medicine, University of Kelaniya, Kelaniya, Sri Lanka; 3 Department of Microbiology, Immunology and Pathology, Colorado State University, Fort Collins, Colorado, United States of America; National Cheng Kung University, TAIWAN

## Abstract

Phylogeographic relationships among global collections of the mosquito *Aedes aegypti* were evaluated using the mitochondrial Cytochrome C Oxidase 1 (CO1) and NADH dehydrogenase subunit 4 (ND4) genes including new sequences from Sri Lanka. Phylogeographic analysis estimated that *Ae*. *aegypti* arose as a species ~614 thousand years ago (kya) in the late Pleistocene. At 545 kya an “early” East African clade arose that continued to differentiate in East Africa, and eventually gave rise to three lineages one of which is distributed throughout all tropical and subtropical regions, a second that contains Southeast Asian/Sri Lankan mosquitoes and a third that contains mostly New World mosquitoes. West African collections were not represented in this early clade. The late clade continued to differentiate throughout Africa and gave rise to a lineage that spread globally. The most recent branches of the late clade are represented by South-East Asia and India/Pakistan collections. Analysis of migration rates suggests abundant gene flow between India/Pakistan and the rest of the world with the exception of Africa.

## Introduction

Viruses transmitted by mosquitoes have become one of the primary contributors to human disease, globally. These include Dengue virus (DENV), Chikungunya virus (CHIKV), Yellow fever virus and Zika virus. Dengue is currently regarded as the most important arboviral disease in the world with approximately 50% of the worlds’ population living in dengue endemic countries. The public health impact of DENV, CHIKV, Yellow fever virus and Zika virus has increased dramatically over the years with both diseases spreading to new areas. *Aedes aegypti*, is the main vector of these viruses and continues to cause a significant amount of human morbidity and mortality worldwide [1, 2].

Dengue is currently estimated to occur in 128 countries worldwide. Regional distribution of dengue shows that it is an aggravated and continuous problem in south and Southeast Asia and Central and South America. Major dengue epidemics have been occurring in Asian and Southeast Asian countries following World War II, which have turned in to pandemics recently [2]. In the Americas, although the control campaign by the Pan American Health Organization (PAHO) restricted the epidemics till 1970s, the suspension of the campaign has reinvested the region to hyper endemic levels of dengue cases [2]. In Sri Lanka, dengue has become a serious arboviral disease with an alarming increase in the number of reported cases. In 2017, 185,688 dengue cases were reported to health authorities with 45% of the cases being reported from the western province, the main commercial province in Sri Lanka [3]. This is 4.7-fold higher than the average number of cases for the same time period between 2010 and 2016 [3]. Although the numbers have decreased in year 2018 with 51,586 recorded dengue cases [3], drought conditions with low rainfall prevailed throughout the year. *Aedes aegypti* is considered as the primary vector of DENVs in Sri Lanka and was first reported in Sri Lanka during 1930s [4].

The global expansion of dengue and other arboviruses was preceded by the global spread of their main vector *Ae*. *aegypti*. *Aedes aegypti* arose as a species in Africa where it differentiated into two subspecies. In Africa, *Ae*. *aegypti formosus* (*Aaf*) has a dark cuticle, no scales on the first abdominal tergite, and lives in forested habitats where it oviposits in natural containers (e.g. tree holes). Adults probably occupied the forest canopy while they fed on non-human primates. Outside of Africa, populations generally consist of the subspecies *Ae*. *aegypti aegypti* (*Aaa*) with a light tan cuticle, scales on the first abdominal tergite, and strong anthropophilic habits [5, 6]. The subspecies eventually adapted to feeding on humans and breeding in artificial containers (e.g. water jars and barrels, and tires). In this way *Aaa* probably colonized the tropical and subtropical regions of the world through shipping trade during the 17^th^—20^th^ centuries [6]. Increasing globalization and the ability to adapt to human environments have enhanced the worldwide spread and establishment of *Aaa* [7], an invasive mosquito that has become a very important arbovirus vector in Asia during the last few years due to the increasing number of dengue cases. Although the two subspecies were originally defined based on differences in coloration and scales on the first abdominal tergite [5, 8] genetic studies have revealed that the West African populations that have these pale scales appear to be genetically more similar to *Aaf* populations than *Aaa* from elsewhere in the tropics [9–11].

Many genetic studies using mitochondrial genes and nuclear genes have evaluated the global colonization history of the *Ae*. *aegypti* subspecies. The earliest studies [12–14] examined the genetic structure and vector competence of *Ae*. *aegypti* using biochemical markers and indicated that global collections fell into two genetic groups. A new world group contained *Aaa* populations from East Africa, South America and the Caribbean and was thought to be derived from East Africa. The second group contained *Aaa* populations from Asia and Southeastern U.S and has a basal branch containing *Aaf* from both East and West Africa. This same pattern has been noted in more recent genetic studies [15–20]. Studies carried out using mitochondrial and nuclear DNA markers have also confirmed the existence of two clades. A study carried out using NADH dehydrogenase subunit 4 (ND4) mitochondrial DNA sequence data from populations of *Ae*. *aegypti* from Senegal, West Africa and East Africa, identified two clades [15], West Africa basal clade and the East Africa derived clade. But these also did not correspond to *Aaa* and *Aaf*. More recent studies based on nuclear markers have used microsatellites [16], a Single Nucleotide Polymorphism (SNP) chip [17, 18] and exon enriched whole genome sequencing [19, 20]. Bennett et al., 2016 and Gloria-Soria et al., 2016 applied Approximate Bayesian Computation (ABC) to both nuclear and mitochondrial markers [7, 16]. ABC supported a demographic model of lineage diversification, historical admixture and recent population structuring. These studies suggested that because *Aaf* are dependent on forests, the effects of forest fragmentation and expansion generated by Pleistocene climatic change may have been a factor in early diversification of populations. Human movement across Africa also probably facilitated more recent divergences. In agreement with other studies Bennett et al., 2016 and Gloria-Soria et al., 2016 [7, 16] noted a decreasing genetic diversity cline consistent with cumulative bottlenecks as *Ae*. *aegypti* was shipped to the Americas and later to Asia. These studies all confirm the existence of a clade at the base of the tree containing *Aaf*, with a second clade derived from the first that contains mosquitoes from East and West Africa. Two genetic groups of *Ae*. *aegypti* in Colombia [21] had haplotypes from both basal and derived clades. This study [21] also revealed that the populations with relatives from the derived clade exhibited insecticide resistance while the population with relatives from the basal clade did not. Studying the genetic differentiation of two lineages is epidemiologically important as populations from different origins vary in vector competencies for arboviruses and insecticide resistance [9, 22, 23].

Given the importance of mitochondria as the primary energy metabolic hub in cells, mitochondrial genes have become instrumental for reconstructing evolutionary relationships within species compared to other genetic markers [24, 25]. Specifically, they are maternally inherited, single copy, non-recombining and abundant [24]. Mitochondria are the site at which the oxidative phosphorylation (OXPHOS) pathway for production of adenosine triphosphate (ATP) takes place. Four of the five complexes in OXPHOS are encoded by mitochondrial genes, NADH dehydrogenase (complex I), cytochrome bc1 complex (complex III), cytochrome c oxidase (complex IV), and ATP synthase (complex V). Thus, mitochondrial protein encoding genes play an important role in the energy metabolism of insects [26]. The mitochondrial genes encoding COI and ND4 are used as the molecular markers in this study. Cytochrome oxidase I (COI) gene possesses special characteristics which make it suitable as a molecular marker for evolutionary studies. It is the largest protein coding gene in the metazoan mitochondrial genome which enables one to amplify and sequence many more characteristics within the same functional complex. It also contains a mix of highly conserved and variable regions so closely associated together thus making COI gene particularly useful for evolutionary studies [27].

The present study is an attempt to estimate the phylogenetic relatedness of Sri Lankan *Ae*. *aegypti* relative to collections from other countries and to estimate a global phylogeographic history for the species. Due to the strategic location in the Indian Ocean, Sri Lanka may be playing a significant role in the distribution of *Ae*. *aegypti* mosquitoes and the four dengue virus serotypes (DENV1-4) in the world. We compile and analyze 546 CO1 and 67 ND4 *Ae*. *aegypti* sequences, including sequences available in GenBank and new CO1 and ND4 sequences from Sri Lanka, a country for which there is little data on the population genetics of *Ae*. *aegypti*.

## Materials and methods

**Fig 1:**
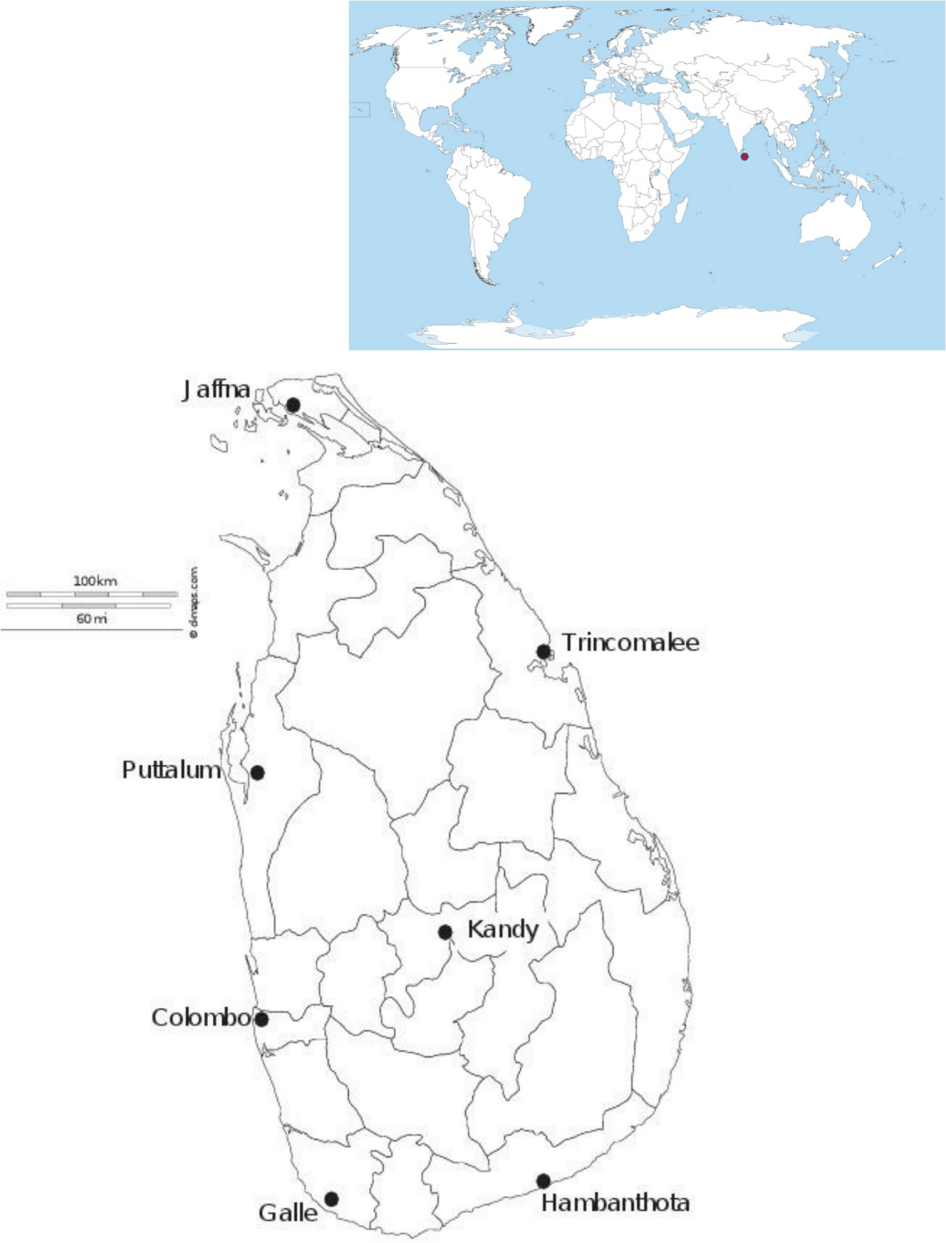
Map of sampling sites in Sri Lanka. The red dot on the global map indicates the geographical position of Sri Lanka in the world.

Adults and larvae of *Ae*. *aegypti* were collected from January 2013 –December 2015 in seven districts in Sri Lanka (Table 1; Fig 1). Permission to collect mosquito samples were obtained from respective Medical Officer of Health (MOH) offices of the study district. Samples were collected using BG-Sentinel adult mosquito traps and ovitraps. Collected larvae were reared to adults and killed by freezing. All adults were morphologically identified to species using standard taxonomic keys [28]. Identified adults were preserved by desiccation with silica gel. For the extraction of DNA, a maximum of 5 individuals per trap were used to avoid over-sampling of siblings. DNA was extracted from individual adults using a Phenol/Chloroform method [29]. Extracted DNA was stored at -80°C in 100 μL TE buffer (10 mM Tris, 1 mM EDTA, pH 8.0). The polymerase chain reaction (PCR) for ND4 and CO1 was carried out in an Eppendorf^®^ Thermo-cycler in 25 μL reaction volumes.

**Table 1:** Sampling districts, geographic coordinates and the number of *Ae*. *aegypti* samples analyzed per each mitochondrial region.

Sampling district	Geographic coordinates		Number of samples analyzed	
	Latitude	Longitude		
			CO1	ND4
Colombo	6^0^ 51’ 16”	79^0^ 54’ 11”	20	20
Galle	6^0^ 47’ 00”	79^0^ 58’ 00”	18	14
Hambanthota	6^0^ 01’ 00”	80^0^ 46’ 60”	19	19
Jaffna	9^0^ 40’ 06”	80^0^ 0’ 23”	15	14
Kandy	7^0^ 17’ 47”	80^0^ 38’ 06”	20	21
Puttalum	8^0^ 14’ 00”	79^0^ 46’ 00”	21	20
Trincomalee	8^0^ 37’ 00”	81^0^ 13’ 00”	19	20

A 349 bp region of the ND4 gene was amplified [21]. Each ND4 reaction consisted of template DNA (2 ng/μL), 1 x PCR Buffer (Promega, USA), 2.0 mM MgCl_2_, 200 μM dNTPs, 0.2 mM of reverse and forward primer and 1 U of *Taq* polymerase (Promega, USA). The reaction conditions entailed an initial denaturation at 94°C for 5 minutes, 35 cycles of 94°C for 30 seconds, 50°C for 30 seconds and 72°C for 30 seconds with a final extension at 72°C for 5 minutes.

A 460 bp region of the mitochondrial CO1 gene was amplified [30]. The CO1 reaction mixture was the same as the ND4 mixture except that it contained 1.5 mM of MgCl_2_ and reaction conditions differed in having an initial denaturation of 94°C for 4 minutes, followed by 40 cycles of denaturation at 95°C for 40 seconds, annealing at 45°C for 1 minute and extension at 68°C for 1 minute, with a final extension of 72°C for 10 minutes. Amplified ND4 and CO1 products were run on a 1.5% agarose gel and the positive products were sent to Macrogen Inc. Korea for sequencing.

Genetic variability in ND4 and CO1 were evaluated by examining the number of substitutions, haplotypes (*h*), nucleotide diversity (*π*) [31], average number of nucleotide differences, k [32] and Fu and Li’s F* [33]. Significantly negative F* indicate an excess of low-frequency variants indicative of population expansion, weak negative selection or positive selection. Significantly positive F* indicate an excess of intermediate-frequency alleles associated with population bottlenecks, breeding structure and/or balancing selection. Diversity at synonymous substitution sites (*π*_*s*_*)* and a replacement substitution sites (*π*_*a*_) were calculated as were their ratio w = (*π*_*a*_) */*(*π*_*s*_) using DnaSP v.5.10 [34]. The sequences used in analysis of the ND4 were all haplotypes recorded previously [15].

To assess the phylogenetic relationship of Sri Lankan *Ae*. *aegypti* to global collections, both ND4 and CO1 sequences from GenBank and Sri Lanka were aligned using CLUSTALW (www.genome.jp/tools/clustalw/) and visually inspected to ensure correct alignment along codons. Any sites exhibiting two or more alternate nucleotides were removed because they were likely to represent nuclear mtDNA (NUMTs) which are abundant in the *Ae*. *aegypti* genome [35, 36].

Sixty-seven sequences of the *Ae*. *aegypti* ND4 gene were aligned which included 27 sequences from GenBank and 40 sequences from Sri Lanka. Six *Aedes* species were included as outgroups (S1 Table). RAxML v.8.000 [37] was used to estimate Maximum Likelihood (ML) trees with bootstrap analysis. The bootstrap node support was estimated with 1,000 pseudoreplicates and the resulting tree was edited using TreeGraph2 (v. 2.0.47) [38]. The support for each labelled branch in the MCC phylogeny was placed above the branch. Branch lengths and their 95% HPD were placed over all labelled nodes.

GenBank contains 546 *Ae*. *aegypti* CO1 sequences and 363 of these contain the same 460 bp region of CO1 as in the current study [39–43]. Sequences from laboratory strains were excluded. All aligned CO1 sequences were compared using RAxML v.8 to identify identical haplotypes and 142 of the 363 sequences were unique (S2 Table). These sequences were partitioned into eight geographic groupings: West Africa (18 sequences), East Africa (38 sequences), India and Pakistan (17 sequences), Sri Lanka (14 sequences), Southeast Asia (13 sequences), South Pacific (6 sequences), North America (6 sequences) and South America (30 sequences). Two sequences (MG242505, HQ693081) from *Ae*. *mascarensis* were included as outgroups since this is considered to be one of the most recent ancestors of *Ae*. *aegypti* [44, 45].

Phylogeographic analysis followed the Bayesian phylogeographic model implemented in BEAST2 [46] v. 2.4.8. The alignment file was converted to an *.xml file using BEAUti following the example provided by Taylor (https://math.la.asu.edu/~jtaylor/teaching/ICEMR2014/geographical_analysis.pdf). Sequences were partitioned into the 8 geographic regions as described above: The Site Substitution model was HKY [47] with the substitution rate set to 1.0, the gamma category count set to 8, the estimated shape parameter set to 1, frequencies were empirical and the estimated Kappa value set to 10. A strict clock model was used. The Clock Rate was set to 3.54 x 10^−8^/site/year [48]. Ten percent of the chains were discarded as burn in. Priors were set to a coalescent constant population model, the gamma shape parameter was set to exponential with a mean of 1, the kappa value was assumed to follow an exponential distribution with a mean of 10, the nonzero states were assumed to follow a Poisson distribution with the lambda set to 0.693 and the offset set to 7, the “popSize” parameter was set to 1/X, the variable “relativeGeorates” were assumed to follow an exponential with a mean of 1.0 and the relative trait clock rate was assumed to follow an exponential with a mean of 1000. This dataset was analyzed with a chain length of 40 million after initial trials with shorter chains failed to produce Effective Sample Sizes (ESS) > 200 for most variables.

Tracer (vers.1.6) (http://tree.bio.ed.ac.uk/software/tracer/) was used to calculate the posterior probability that the migration rate (“rateIndicator”) is positive between a pair of geographic regions x and y. A large probability indicates direct migration between x and y while a small posterior probability suggests barriers to gene flow. The strength of support for a particular pair of regions was also assessed by calculating its Bayes Factor (BF) as the ratio of the posterior and prior probabilities following (https://math.la.asu.edu/~jtaylor/teaching/ICEMR2014/geographical_analysis.pdf). If D is the number of regions and p_i_ is the posterior probability that the migration rate is positive then the prior probability (q_x,y_) is

$${\text{q}}_{\text{x},\text{y}}=\frac{\text{ln}\left(2\right)+\left(\text{D}-1\right)}{\left(\frac{\text{D}\left(\text{D}-1\right)}{2}\right)}$$ (1)

There are 8 regions and so q_x,y_ = 0.2748.

$$\text{BFxy}=\frac{\text{px},\text{y}}{\left(1-\text{px},\text{y}\right)}/\frac{\text{qx},\text{y}}{\left(1-\text{qx},\text{y}\right)}$$ (2)

Rates with Bayes factors greater than 3 were considered to be well supported. TreeAnnotator v.2.4.3 was used to create the maximum clade credibility tree with each taxon identified.

## Results

### Variability in different geographic regions

Table 2 shows genetic diversity measures at both genes. Diversity was relatively low in West Africa but was similar in all other regions including Sri Lanka. Fu and Li’s F* measures were significantly negative (excess of singletons) when considering all regions. This is probably indicative of population expansion. ND4 sequences that occurred in common among all or some of the regions were analyzed separately and appear as “Global” in Table 2. These had a significantly positive Fu and Li’s F* indicative of an excess of intermediate-frequency haplotypes associated with population bottlenecks, genetic structure and/or balancing selection. In all cases w is small and close to zero suggesting purifying selection acting in both genes.

**Table 2:** Genetic variability in ND4 and CO1 were evaluated by examining the number of substitutions (S), haplotypes (h), nucleotide diversity (π) [31], average number of nucleotide differences, k [32] and Fu and Li’s F* [33].

Gene & Region	Sample Size	S	h	Π	k	Fu & Li’s F*	π(s)	π(a)	w
**ND4**									
**All Collections**	157	59	136	0.0215	7.5050	-3.0598**	0.0689	0.0051	0.0746
**West Africa**	19	20	19	0.0119	4.1404	-1.0688	0.0403	0.0027	0.0663
**East Africa**	8	18	7	0.0212	7.3929	0.3944	0.0763	0.0033	0.0429
**Sri Lanka**	40	17	24	0.0198	6.9231	1.3833	0.0536	0.0065	0.1214
**Southeast Asia**	26	26	26	0.0203	7.0985	-0.4817	0.0711	0.0039	0.0551
**North America**	15	23	15	0.0262	9.1333	-0.0321	0.0839	0.0076	0.0905
**South America**	35	26	34	0.0206	7.2034	-0.5413	0.0711	0.0044	0.0613
**Global**	14	15	14	0.0199	6.9560	1.8584**	0.0723	0.0030	0.0416
**CO1**									
**All Collections**	142	81	103	0.0153	7.0244	-3.4919**	0.0153	0.0045	0.2960
**West Africa**	17	12	14	0.0060	2.7647	-0.9844	0.0209	0.0007	0.0311
**East Africa**	36	35	33	0.0148	6.8206	-1.4287	0.0474	0.0031	0.0654
**India/Pakistan**	17	29	15	0.0108	4.9559	-2.1729	0.0266	0.0051	0.1918
**Sri Lanka**	14	17	14	0.0140	6.4286	-0.2164	0.0417	0.0040	0.0961
**Southeast Asia**	13	16	9	0.0132	6.0513	1.0384	0.0399	0.0036	0.0895
**South Pacific**	5	5	5	0.0052	2.4000	0.0000	0.0197	0.0000	0.0000
**North America**	6	13	5	0.0130	6.0000	0.4555	0.0394	0.0036	0.0903
**South America**	31	37	27	0.0157	7.2409	-1.8038	0.0376	0.0079	0.2114

Diversity at synonymous substitution sites (πs) and a replacement substitution sites (πa) were calculated as were their ratio w = (πa) /(πs) using DnaSPv5.10 [34]. ** indicates probabilities that absolute F* values = 0 is < 0.01. The taxa used in analysis of the ND4 were all haplotypes in S1 Fig [15]. All Collections refers to all collections combined. Global refers to haplotypes that appear in one or more regions.

### Phylogenetic analysis of ND4 markers

**Fig 2:**
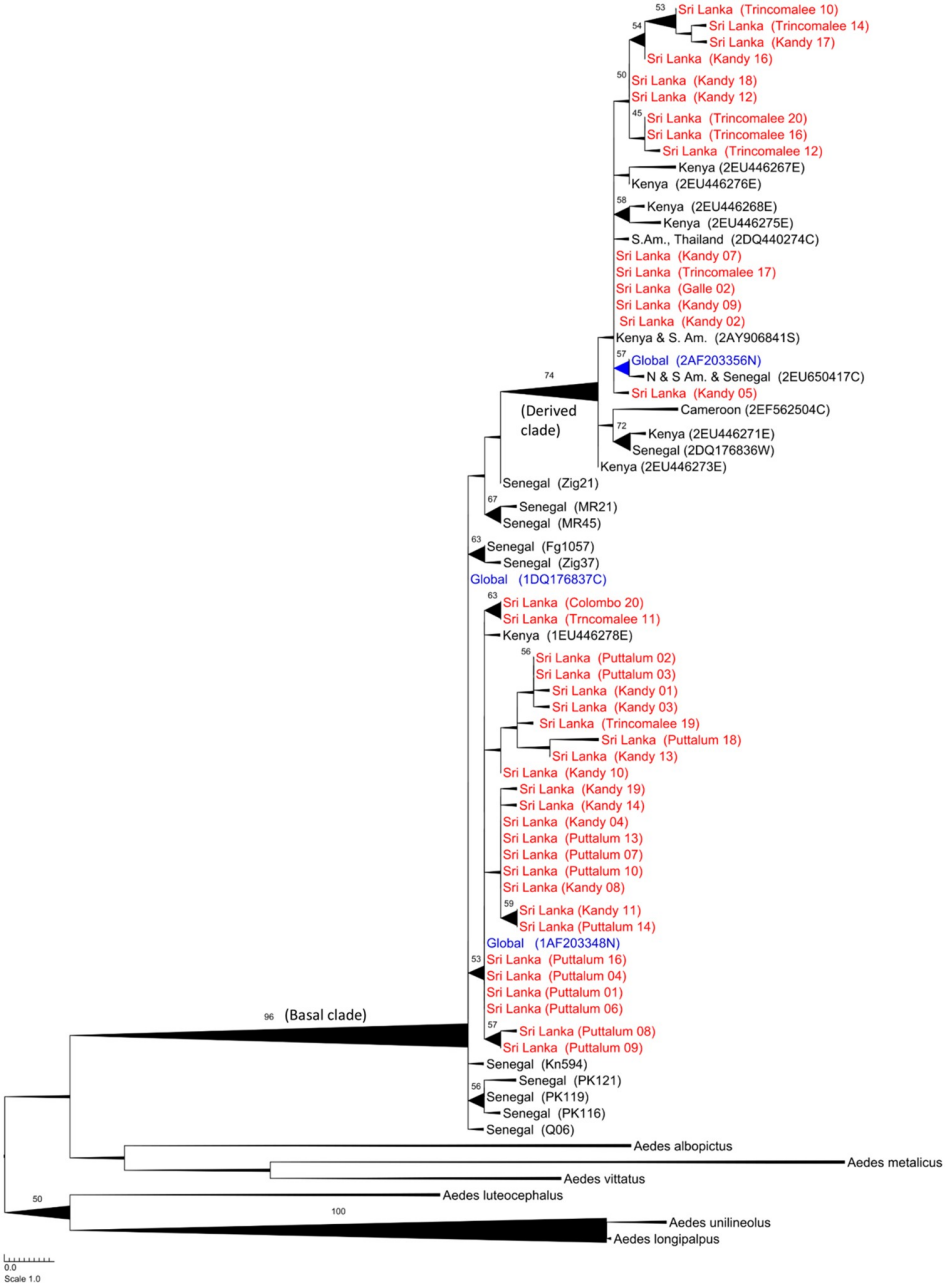
Bootstrapped Maximum Likelihood tree derived using the ND4 gene. The “basal” clade has bootstrap support of 96% and contains the West African collections at its base. The “derived” clade (bootstrap support = 74%) has both East and West African collections at its base, global collections (AF203356) and many East African sequences from Kenya. All Sri Lankan collections appear in red and are present in both clades. Information in parentheses indicate the collection site and its unique identification number in the aligned dataset. The thickness of lines is proportional to the bootstrap support. Maximum likelihood branch lengths are proportional to the number of nucleotide substitutions per site.

Fig 2 is the bootstrapped ML tree derived using the ND4 gene. It has a “basal” clade (bootstrap support = 96%) containing all ND4 *Ae*. *aegypti* sequences but including West African collections at the base, global collections (AF203348, DQ176837) and the East African sequence from Kenya (EU446278). It also has a “derived” clade (bootstrap support = 74%) that has both East and West African collections at its base, global collections (AF203356) and many East African sequences from Kenya. Thus, Sri Lankan populations consist of the same two previously designated West (basal) and East (derived) African clades. The paucity of ND4 sequences on the web and the absence of a molecular clock for ND4 obviated more intensive phylogeographic analyses. Within this clade, there is only moderate resolution of clades with support values never exceeding 75.

### Phylogenetic analysis of CO1 markers

**Fig 3:**
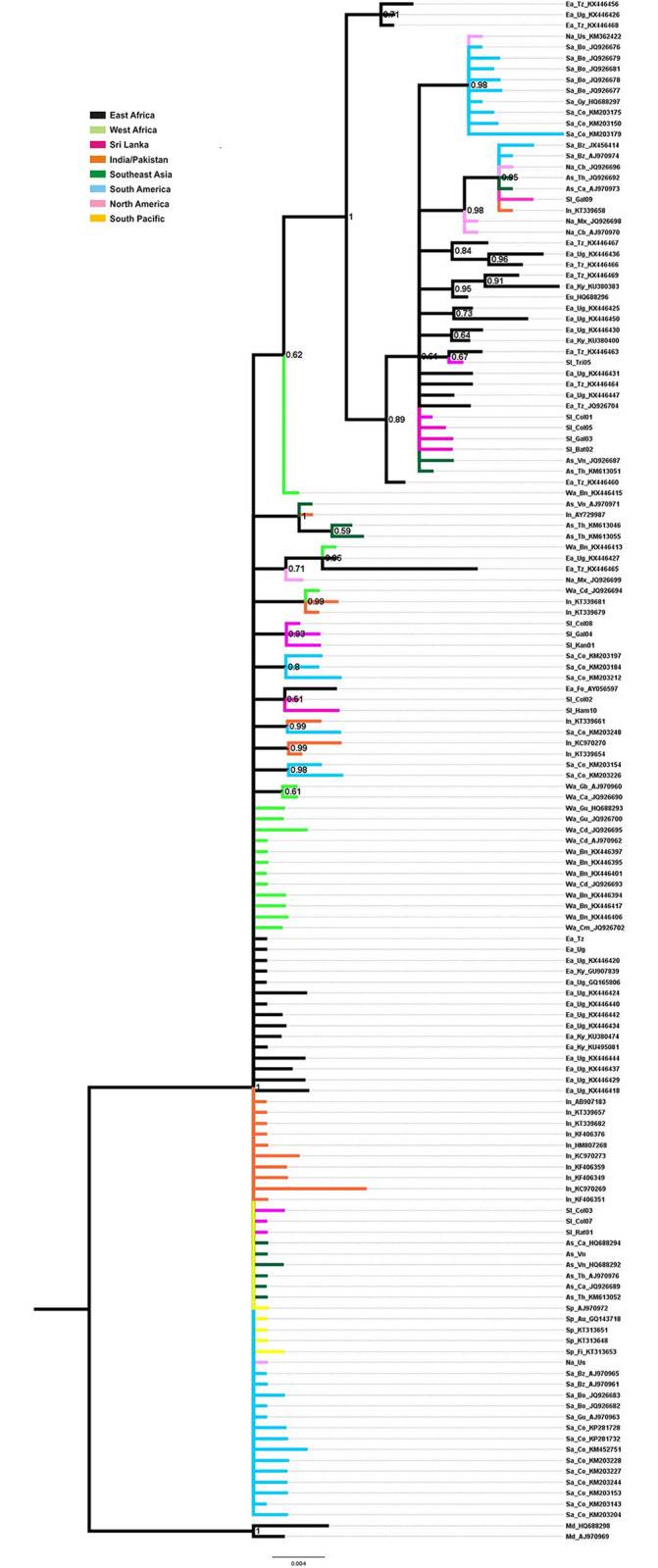
Bayesian phylogenetic tree of relationships of *Ae*. *aegypti* based on CO1 gene. The support values written on the branches correspond to posterior probabilities. The branch lines are colored by geographic distribution of the corresponding haplotypes.

Fig 3 is the Mrbayes tree derived using the CO1 gene. The tree has a main clade (posterior probability = 1) containing both East and West African sequences. The derived clade mainly contains East African collections. The nodes arising from this clade gave rise to Sri Lankan, South American and Southeast Asian collections.

**Fig 4:**
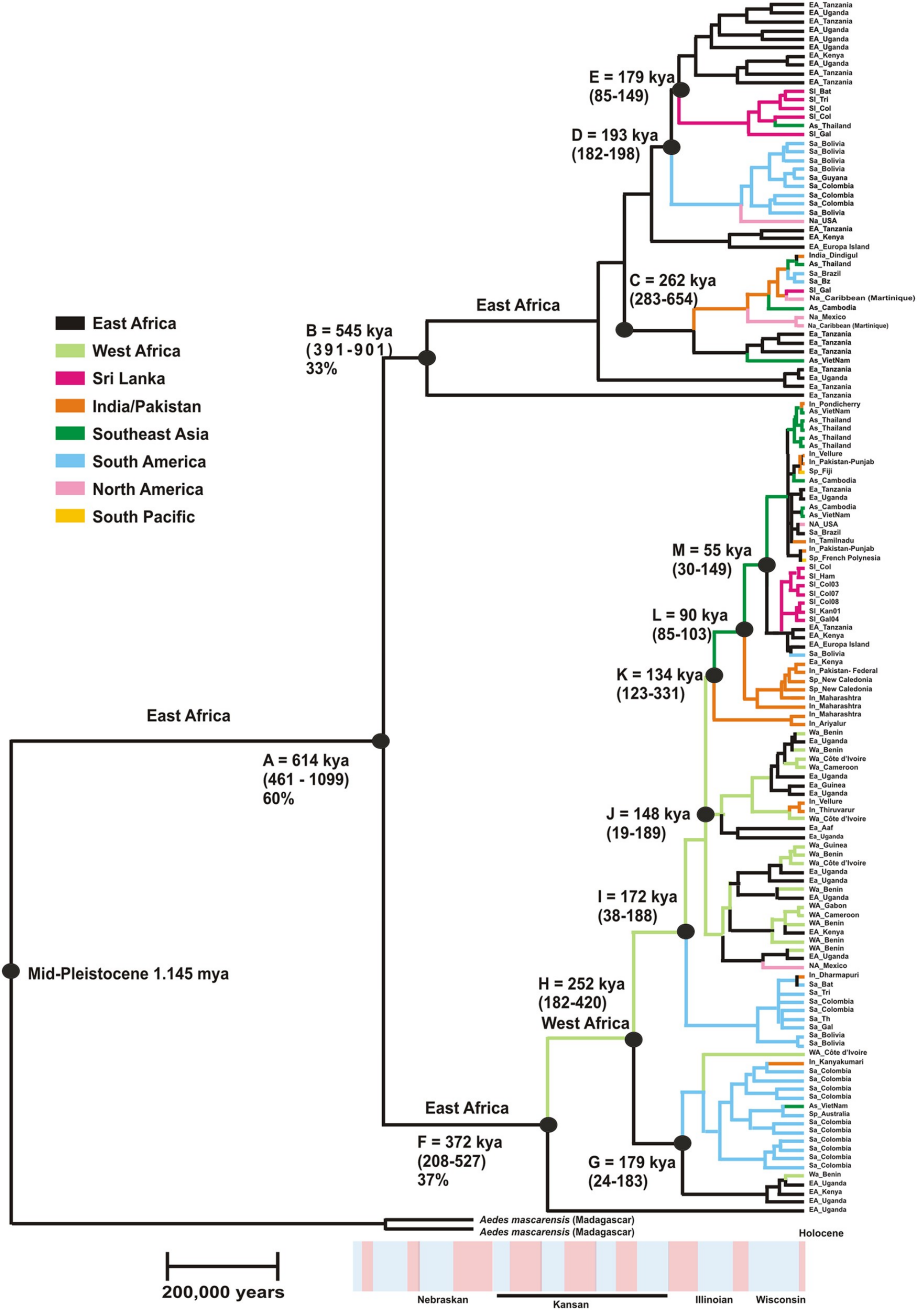
Phylogeographic analysis of the CO1 gene followed the Bayesian phylogeographic model implemented in BEAST2 [46] v. 2.4.8. The alignment file was converted to an *.xml file using BEAUti following the example provided by Taylor (https://math.la.asu.edu/~jtaylor/teaching/ICEMR2014/geographical_analysis.pdf). Sequences were partitioned into 8 geographic regions. A scale corresponding to 200,000 years is provided at the base. The identity of nodes appears in the text.

Fig 4 is the coalescent tree derived using the CO1 gene. The A clade arose 614 thousand years ago (kya) in East Africa. The Node B clade corresponds to the previously designated “derived” clade and it began to diversify 545 kya in East Africa. The East African Node C gave rise to a clade that has spread globally. East African Node D gave rise to a clade that eventually spread to the New World while clade E spread to Sri Lanka and Southeast Asia. One East African lineage arising from Node E arose 179 kya and has diversified in East Africa.

The Node F clade has a support value of 37 (previously designated “basal” [15]) did not began to diversify until 372 kya, ~173 kya later than node B. Notice that no West African haplotypes appear in the Node B clade. A split (Node H) occurred in West Africa 252 kya. One of these led to East African clade arising from Node G. The lower and upper branch of Node G contains one West African haplotype each.

Both observations indicate that introgression into West Africa began from 252 kya (Node H) to 372 kya. Node G also gave rise to lineages that eventually spread to South America. Subsequent lineages (Nodes I and J) arising from the West African Node H continued to diversify in West Africa and East Africa. Node I gave rise to a lineage that eventually spread to South America. Node J subsequently gave rise to two lineages that appear to have remained in East and West Africa. One of these contains a Mexican haplotype and the other an Indian/Pakistan isolate. Node K (134 kya) appears to mark a time when West African lineages began to spread out of Africa with one lineage ending up in India, Sri Lanka and the other shifting toward Southeast Asia (Node L) with one lineage ending up in India/Pakistan and the South Pacific. Southeast Asia Node K contains one lineage that re-colonized East Africa and from there colonized Sri Lanka and eventually ended up in South America. The other clade arising from Node M produced haplotypes that occur globally today.

The ages represented near the tips seem unreasonably old. For instance, multiple clades comprised exclusively of New World populations have common ancestors more than 50 kya, which is far outside the current hypotheses on when *Aedes aegypti* escaped Africa. However, the 95% HPD for almost all terminal nodes includes a lower value of zero.

### Migration analysis

Analysis of the rate Indicator variables in BEAST appears in Table 3. All rate Indicators with Bayes Factors ≥ 3 are underlined and bolded. This analysis indicates a high rate of gene flow between the India/Pakistan region and all other regions except Africa and, curiously Sri Lanka. There are also high rates of gene flow between East and West Africa and between Sri Lanka and Southeast Asia.

**Table 3:** A) Tracer analysis of the posterior probability, likelihood, Prior probability, tree likelihood and average tree height. B) Tracer analysis of posterior probability that the migration rate (*rateIndicator*) is positive between a pair of geographic regions x and y.

**Summary Statistic**		**mean**	**Std.error**	**ESS**	
(A) posterior		-4397.5	2.17	183.31	
Likelihood		-2070.8	1.24	132.13	
Prior		-2326.7	1.26	345.01	
treeLikelihood		-1874.3	1.34	101.39	
TreeHeight		1506600.0	14095.50	715.40	
**(B) RateIndicator**					
**Region x**	**Region y**	**Mean**	**Std.error**	**ESS**	**BF**
Sri Lanka	India/Pakistan	0.3330	0.0236	398	1.3
Sri Lanka	East Africa	0.0533	0.0110	415	0.1
Sri Lanka	West Africa	0.0300	0.0060	815	0.1
Sri Lanka	SouthEast Asia	0.5716	0.0280	311	3.5
Sri Lanka	South Pacific	0.0810	0.0110	617	0.2
Sri Lanka	North America	0.1343	0.0142	579	0.4
Sri Lanka	South America	0.0277	0.0059	774	0.1
India/Pakistan	East Africa	0.4151	0.0515	92	1.9
India/Pakistan	West Africa	0.4939	0.0454	121	2.6
India/Pakistan	SouthEast Asia	0.9401	0.0087	737	41.4
India/Pakistan	South Pacific	0.5327	0.0235	451	3.0
India/Pakistan	North America	0.5982	0.0214	525	3.9
India/Pakistan	South America	0.6792	0.0337	191	5.6
East Africa	West Africa	0.9545	0.0251	69	55.4
East Africa	SouthEast Asia	0.1265	0.0183	329	0.4
East Africa	South Pacific	0.0289	0.0068	611	0.1
East Africa	North America	0.0555	0.0123	349	0.2
East Africa	South America	0.0344	0.0086	454	0.1
West Africa	SouthEast Asia	0.0788	0.0100	725	0.2
West Africa	South Pacific	0.0455	0.0086	581	0.1
West Africa	North America	0.0954	0.0151	379	0.3
West Africa	South America	0.1221	0.0296	122	0.4
SouthEast Asia	South Pacific	0.5061	0.0242	427	2.7
SouthEast Asia	North America	0.3208	0.0189	610	1.2
SouthEast Asia	South America	0.1410	0.0236	218	0.4
South Pacific	North America	0.3074	0.0189	598	1.2
South Pacific	South America	0.0677	0.0134	353	0.2
North America	South America	0.3230	0.0324	208	1.3

A large probability indicates direct migration between x and y while a small posterior probability would suggest barriers to gene flow. The strength of support for a particular pair of regions was also assessed by calculating its Bayes Factor (BF) as the ratio of the posterior and prior odds (Eq 2). Rates with Bayes factors greater than 3 appear as underlined bold letters. (ESS–Effective sample size, Std.error–Standard error).

## Discussion

The present study analyzed mitochondrial genetic diversity and phylogeographic relationships of *Ae*. *aegypti* mosquitoes collected from seven districts in Sri Lanka. The study revealed a high genetic diversity for CO1 marker which might be due to high levels of gene flow observed in the study between Sri Lanka and Southeast Asia. A high level of gene flow exists between India /Pakistan region and Southeast Asia, South Pacific and North and South America. This would inevitably lead to population mixing. As Sri Lanka is an island and acts as a main harbor for commercial and human transport it may be playing a vital role in the observed gene flow.

Two mitochondrial clades have been reported previously that broadly represent East and West Africa, but this was based only on the ND4 gene [15]. Furthermore, no time frame could be estimated for these clades because a calibrated molecular clock rate is not available for the arthropod ND4 gene. However, an independent molecular clock has been estimated for CO1 [48] and at least seven large datasets for CO1 in *Ae*. *aegypti* have appeared [7, 21, 39–43]. Some of the problems associated with studies of the mitochondrial genome in *Ae*. *aegypti* have been recently summarized [5].

Approximate Bayesian Computation (ABC) [49] has been applied to nucleotide sequences from four nuclear and one mitochondrial marker to assess phylogeographic relationships among *Ae*. *aegypti* collections principally in Africa [7]. Their analyses showed that a model wherein *Ae*. *aegypti* originated in Africa involving Pleistocene lineage diversification and historical admixture had the best fit in the ABC.

Marine records of African climate variability document a shift toward more arid conditions beginning 2.8 million years ago (mya), resulting from cold North Atlantic sea-surface temperatures caused by the beginning of northern hemisphere glacial cycles [50–53]. The maximum of the penultimate glaciations occurred from 190–150 kya (corresponding with the Illinoian Stage) and conditions in Africa became generally drier than present with deserts extending into North Africa. Then from 150–130 kya, Africa gradually became cooler than present. This was followed by a warmer, moister period that lasted for 15,000 years. During that time deserts became covered with vegetation and there was a great expansion of rain forests. Then from 115–70 kya, there followed a period of cooling and drying that led to a cold, arid maximum (corresponding to the Wisconsin period) followed by a slight moderation of climate until 22 kya. From 115–70 kya conditions became warmer and moister but with an interruption by aridity around 11,000 years ago. A resumption of warm, moist conditions known as the Holocene ‘optimum’ occurred wherein again there was a second great rain forest expansion with vegetation again covering most of the Sahara. From then until the present Africa has become more arid. Relatively brief arid phases (e.g. 8,200 ya.) have punctuated the generally moister early and mid-Holocene conditions. Earlier studies assumed that ancestral *Ae*. *aegypti* were dependent on forests, and that forest fragmentation driven by climatic change may have been responsible for the lineage splitting in the late Pleistocene [7].

In almost all respects the findings of the current study support Bennett’s model wherein *Ae*. *aegypti* originated in Africa and that one or more Pleistocene lineage diversifications occurred. This has been followed by many historical instances of admixture. Differences in time scale between our study and Bennett et al. [7] are likely a result of applying different divergence rates. Bennett et al. [7] used 2.69 x 10^−8^/site/year which is the mitochondrial rate with both the 16S rRNA and CO1 rates combined [48] and 2.3 x 10^−8^/site/year [53]. We instead used 3.54 x 10^−8^/site/year [48] which is the rate for the CO1 alone. Nevertheless, our estimates are correlated with Bennett et al. [7] that lineages diverged at node A 700–3000 kya while we estimate that node A occurred 614 kya.

Our phylogeographic analysis suggests that the Pleistocene lineage diversification(s) probably happened in East Africa. The first East African lineage (Node B) (Fig 3) has remained in East Africa where it continues to diversify (Node E) (Fig 3) and has spread to Sri Lanka and Southeast Asia. Our original designation of Node B as the “derived” lineage [15] was incorrect. Instead the East African lineage evolved 173 kya before the lineage giving rise to the West Africa clade (Node F) and it should therefore be considered as basal. The West Africa clade is the “derived” lineage and it wasn’t until 372 kya that it arrived and began to diversify in West Africa. From there it spread to all global regions examined in the present study.

As with Bennett et al. [7], there are many cases where individuals appear to have been reintroduced into regions of origin. Nodes I and J (Fig 3) arise from West African lineages but contain many sequences from East Africa. Also, nodes K, L, M (Fig 3) arise from Southeast Asian lineages but gave rise to sequences eventually found in East Africa, India / Pakistan, and Sri Lanka. This reflects the admixtures generated by global spread of *Ae*. *aegypti*. The question remains as to whether these results indicate a current high level of gene flow between regions in the invasive range of the mosquito or represent shared ancestral polymorphisms.

It is interesting that both groups of Sri Lanka haplotypes cluster together and have recent origins. The two origins are, one from India/Pakistan and another from mixed clades of mosquitoes derived from East Africa. This appears to be counter intuitive based upon the proximity of Sri Lanka to India and its great distance to East Africa and the fact that most of the India collections come from South India [42]. The analysis of migration rates in Table 3 supports the idea that while there is a great deal of gene flow, among mosquitoes within the India/Pakistan Region there is very little gene flow between India/Pakistan and Africa or Sri Lanka. This analysis also supports the idea that gene flow occurs between Southeast Asian countries and Sri Lanka. Sri Lanka is situated along the key shipping route between the Malacca Straits and the Suez Canal. Approximately 36,000 ships, including 4,500 oil tankers, use the route annually [54]. Thus, Sri Lanka may be playing a major role in the trafficking of DENV, its vector *Ae*. *aegypti* and infected humans from Southeast Asia to the Middle East and Africa.

## Conclusions

*Ae*. *aegypti* appears to have arisen as a species in East Africa in the mid-late Pleistocene probably associated with a shift towards more arid conditions beginning 2.8 million years ago (mya), resulting from cold North Atlantic sea-surface temperatures that caused the beginning of northern hemisphere glacial cycles. Two mitochondrial clades arose in the late Pleistocene, but these subsequently became admixed during periods of warming and the spread of forests. An analysis of migration rates suggests a great deal of gene flow between mosquitoes in the India/Pakistan Region and the rest of the world with the exception of Africa and Sri Lanka. There appears to be abundant gene flow between Southeast Asian countries and Sri Lanka.

## Supporting information

S1 Table Region and country of origin of *Aedes aegypti* NADH dehydrogenase subunit 4 (ND4) gene. The GenBank accession number and publication reference is listed [55–58].
(DOCX)

S2 Table Region and country of origin of *Aedes aegypti* Cytochrome C Oxidase 1 (CO1) gene sequences. The GenBank accession number and publication reference is listed [59–68].
(DOCX)
