# Supplementary material for: Mitochondrial metabolic genes provide phylogeographic relationships of global collections of Aedes aegypti (Diptera: Culicidae)
Source: PLoS One. 2020 Jul 28;15(7):e0235430. doi: 10.1371/journal.pone.0235430 (PMC7386613; doi:10.1371/journal.pone.0235430)
Supplement: S1 Table — The GenBank accession number and publication reference is listed [55–58]. (DOCX) [file pone.0235430.s001.docx]

**S1 Table. Region and country of origin of *Aedes aegypti* NADH dehydrogenase subunit 4 (ND4) gene.** The GenBank accession number and publication reference is listed.

| **Country** | **GenBank accession no.** | **Reference** |
| --- | --- | --- |
| Sri Lanka (Kandy07) | KY476368 | Current MS |
| Sri Lanka (Kandy09) | KY476369 | Current MS |
| Sri Lanka (Kandy05) | KY476370 | Current MS |
| Sri Lanka (Kandy01) | KY476371 | Current MS |
| Sri Lanka (Kandy02) | KY476372 | Current MS |
| Sri Lanka (Kandy03) | KY476373 | Current MS |
| Sri Lanka (Kandy04) | KY476374 | Current MS |
| Sri Lanka (Kandy08) | KY476375 | Current MS |
| Sri Lanka (Kandy10) | KY476376 | Current MS |
| Sri Lanka (Kandy11) | KY476377 | Current MS |
| Sri Lanka (Kandy12) | KY476378 | Current MS |
| Sri Lanka (Kandy13) | KY476379 | Current MS |
| Sri Lanka (Kandy14) | KY476380 | Current MS |
| Sri Lanka (Kandy16) | KY476381 | Current MS |
| Sri Lanka (Kandy17) | KY476382 | Current MS |
| Sri Lanka (Kandy18) | KY476383 | Current MS |
| Sri Lanka (Kandy19) | KY476384 | Current MS |
| Sri Lanka (Puttalum01) | KY476385 | Current MS |
| Sri Lanka (Puttalum02) | KY476386 | Current MS |
| Sri Lanka (Puttalum03) | KY476387 | Current MS |
| Sri Lanka (Puttalum04) | KY476388 | Current MS |
| Sri Lanka (Puttalum06) | KY476389 | Current MS |
| Sri Lanka (Puttalum07) | KY476390 | Current MS |
| Sri Lanka (Puttalum08) | KY476391 | Current MS |
| Sri Lanka (Puttalum09) | KY476392 | Current MS |
| Sri Lanka (Puttalum10) | KY476393 | Current MS |
| Sri Lanka (Puttalum13) | KY476394 | Current MS |
| Sri Lanka (Puttalum14) | KY476395 | Current MS |
| Sri Lanka (Puttalum16) | KY476396 | Current MS |
| Sri Lanka (Puttalum18) | KY476397 | Current MS |
| Sri Lanka (Galle02) | KY496642 | Current MS |
| Sri Lanka (Colombo20) | KY496643 | Current MS |
| Sri Lanka (Trincomalee10) | KY496644 | Current MS |
| Sri Lanka (Trincomalee11) | KY496645 | Current MS |
| Sri Lanka (Trincomalee12) | KY496646 | Current MS |
| Sri Lanka (Trincomalee14) | KY496647 | Current MS |
| Sri Lanka (Trincomalee16) | KY496648 | Current MS |
| Sri Lanka (Trincomalee17) | KY496649 | Current MS |
| Sri Lanka (Trincomalee19) | KY496650 | Current MS |
| Sri Lanka (Trincomalee20) | KY496651 | Current MS |
| Cameroon | EF562502 | Unpublished |
| Kenya | EU446267 | Unpublished |
| Kenya | EU446276 | Unpublished |
| Kenya | EU446273 | Unpublished |
| Kenya | EU446271 | Unpublished |
| Brazil | DQ176836 | Unpublished |
| Brazil | AY906841 | [55] |
| USA | DQ440274 | [56] |
| Kenya | EU446268 | Unpublished |
| Kenya | EU446275 | Unpublished |
| Mexico | AF203356 | [57] |
| Brazil | EU650417 | [58] |
| Cameroon | EF562504 | Unpublished |
| Mexico | AF203348 | [57] |
| Kenya | EU446278 | Unpublished |
| Brazil | DQ176837 | Unpublished |
| Senegal(Ouakam-06) | JX427521 | [15] |
| Senegal(Ziguinchor-21) | JX427524 | [15] |
| Senegal(Ziguinchor-37) | JX427525 | [15] |
| Senegal(Fongolimbi-1057) | JX427511 | [15] |
| Senegal(Koungheul-594) | JX427515 | [15] |
| Senegal(Mont Rolland-21) | JX427516 | [15] |
| Senegal(Mont Rolland-45) | JX427517 | [15] |
| Senegal(PK10 Forest-116) | JX427518 | [15] |
| Senegal(PK10 Forest-119) | JX427519 | [15] |
| Senegal(PK10 Forest-121) | JX427520 | [15] |
| Brazil (Rio de Janeiro) | EF153759 | Unpublished |
| Senegal(PK10)*Aedes metallicus* | JX427526 | [15] |
| Senegal(PK10)*Aedes vittatus* | JX427529 | [15] |
| Senegal(PK10)*Aedes luteocephalus* | JX427527 | [15] |
| Senegal (PK10)*Aedes unilineatus* | JX427530 | [15] |
| Senegal (PK10)Aedes *longipalpis* | JX427528 | [15] |
| Brazil *Aedes albopictus* | EF153761 | Unpublished |
