# Supplementary material for: Mitochondrial metabolic genes provide phylogeographic relationships of global collections of Aedes aegypti (Diptera: Culicidae)
Source: PLoS One. 2020 Jul 28;15(7):e0235430. doi: 10.1371/journal.pone.0235430 (PMC7386613; doi:10.1371/journal.pone.0235430)
Supplement: S2 Table — The GenBank accession number and publication reference is listed [59–68]. (DOCX) [file pone.0235430.s002.docx]

**S2 Table. Region and country of origin of *Aedes aegypti* Cytochrome C Oxidase 1 gene (CO1) sequences.** The GenBank accession number and publication reference is listed.

| **Region** | **Country** | **GenBank accession no.** | **Reference** |
| --- | --- | --- | --- |
| West Africa | Guinea | HQ688293 | [59] |
| West Africa | Guinea | JQ926700 | [41] |
| West Africa | Côte d'Ivoire | JQ926695 | [41] |
| West Africa | Côte d'Ivoire | AJ970962 | [40] |
| West Africa | Benin | KX446397 | [7] |
| West Africa | Benin | KX446395 | [7] |
| West Africa | Gabon | AJ970960 | [40] |
| West Africa | Cameroon | JQ926690 | [41] |
| West Africa | Benin | KX446413 | [7] |
| West Africa | Benin | KX446401 | [7] |
| West Africa | Côte d'Ivoire | JQ926693 | [41] |
| West Africa | Benin | KX446394 | [7] |
| West Africa | Benin | KX446415 | [7] |
| West Africa | Benin | KX446417 | [7] |
| West Africa | Benin | KX446406 | [7] |
| West Africa | Côte d'Ivoire | JQ926694 | [41] |
| West Africa | Cameroon | JQ926702 | [41] |
| West Africa | Guinea | AJ970963 | [40] |
| East Africa | Tanzania | KX446455 | [7] |
| East Africa | Uganda | KX446451 | [7] |
| East Africa | Formosus | AY056597 | [60] |
| East Africa | Uganda | KX446420 | [7] |
| East Africa | Kenya | GU907839 | [61] |
| East Africa | Uganda | GQ165806 | [62] |
| East Africa | Uganda | KX446425 | [7] |
| East Africa | Kenya | KU380400 | [63] |
| East Africa | Tanzania | KX446467 | [7] |
| East Africa | Tanzania | KX446463 | [7] |
| East Africa | Uganda | KX446431 | [7] |
| East Africa | Uganda | KX446430 | [7] |
| East Africa | Tanzania | KX446469 | [7] |
| East Africa | Tanzania | KX446468 | [7] |
| East Africa | Uganda | KX446426 | [7] |
| East Africa | Tanzania | KX446466 | [7] |
| East Africa | Tanzania | KX446464 | [7] |
| East Africa | Tanzania | KX446460 | [7] |
| East Africa | Uganda | KX446447 | [7] |
| East Africa | Tanzania | JQ926704 | [41] |
| East Africa | Tanzania | KX446456 | [7] |
| East Africa | Uganda | KX446450 | [7] |
| East Africa | Uganda | KX446436 | [7] |
| East Africa | Kenya | KU380383 | [63] |
| East Africa | Uganda | KX446427 | [7] |
| East Africa | Uganda | KX446424 | [7] |
| East Africa | Uganda | KX446440 | [7] |
| East Africa | Uganda | KX446442 | [7] |
| East Africa | Uganda | KX446434 | [7] |
| East Africa | Kenya | KU380474 | [63] |
| East Africa | Uganda | KX446444 | [7] |
| East Africa | Uganda | KX446437 | [7] |
| East Africa | Uganda | KX446429 | [7] |
| East Africa | Uganda | KX446418 | [7] |
| East Africa | Tanzania | KX446465 | [7] |
| East Africa | Madagascar | HQ688298 | [59] |
| East Africa | Madagascar | AJ970969 | [40] |
| East Africa | Europa Island | HQ688296 | [59] |
| India and Pakistan | India: Dindigul | KT339658 | [42] |
| India and Pakistan | India:Tamilnadu | AB907183 | Unpublished |
| India and Pakistan | India: Vellure | KT339681 | [42] |
| India and Pakistan | India: Kanyakumari | KT339661 | [42] |
| India and Pakistan | India: Dharmapuri | KT339657 | [42] |
| India and Pakistan | India: Viluppuram | KT339682 | [42] |
| India and Pakistan | Pakistan: Punjab | KF406376 | [39] |
| India and Pakistan | India: Thiruvarur | KT339679 | [42] |
| India and Pakistan | India: Thiruvananthapuram | HM807268 | Unpublished |
| India and Pakistan | India: Pondicherry | AY729987 | [64] |
| India and Pakistan | India: Maharashtra | KC970273 | Unpublished |
| India and Pakistan | Pakistan: Federal | KF406359 | [39] |
| India and Pakistan | Pakistan: Punjab | KF406349 | [39] |
| India and Pakistan | India: Maharashtra | KC970270 | Unpublished |
| India and Pakistan | India: Ariyalur | KT339654 | [42] |
| India and Pakistan | India: Maharashtra | KC970269 | Unpublished |
| India and Pakistan | Pakistan: Punjab | KF406351 | [39] |
| Sri Lanka | Colombo01 | KY476354 | Current MS |
| Sri Lanka | Colombo02 | KY476355 | Current MS |
| Sri Lanka | Colombo03 | KY476356 | Current MS |
| Sri Lanka | Colombo05 | KY476357 | Current MS |
| Sri Lanka | Colombo07 | KY476358 | Current MS |
| Sri Lanka | Colombo08 | KY476359 | Current MS |
| Sri Lanka | Galle03 | KY476360 | Current MS |
| Sri Lanka | Galle04 | KY476361 | Current MS |
| Sri Lanka | Galle09 | KY476362 | Current MS |
| Sri Lanka | Batticaloa02 | KY476364 | Current MS |
| Sri Lanka | Batticaloa 01 | KY476363 | Current MS |
| Sri Lanka | Trincomalee05 | KY476365 | Current MS |
| Sri Lanka | Hambanthota10 | KY476366 | Current MS |
| Sri Lanka | Kandy01 | KY476367 | Current MS |
| Southeast Asia | Cameroon | AJ970973 | [40] |
| Southeast Asia | Thailand | JQ926692 | [41] |
| Southeast Asia | Cameroon | HQ688294 | [59] |
| Southeast Asia | VietNam | AJ970967 | [40] |
| Southeast Asia | VietNam | HQ688292 | [59] |
| Southeast Asia | VietNam | JQ926687 | [41] |
| Southeast Asia | Thailand | AJ970976 | [40] |
| Southeast Asia | Cameroon | JQ926689 | [41] |
| Southeast Asia | Thailand | KM613052 | Unpublished |
| Southeast Asia | VietNam | AJ970971 | [40] |
| Southeast Asia | Thailand | KM613051 | Unpublished |
| Southeast Asia | Thailand | KM613055 | Unpublished |
| Southeast Asia | Thailand | KM613046 | Unpublished |
| South Pacific | French Polynesia | AJ970972 | [40] |
| South Pacific | Australia | GQ143718 | [65] |
| South Pacific | Fiji | KT313651 | [66] |
| South Pacific | Tonga | KT313648 | [66] |
| South Pacific | Fiji | KT313653 | [66] |
| South Pacific | Australia | KU495081 | [67] |
| North America | USA | JQ926684 | [41] |
| North America | Caribbean | JQ926696 | [41] |
| North America | USA | KM362422 | [58] |
| North America | Mexico | JQ926699 | [41] |
| North America | Mexico | JQ926698 | [41] |
| North America | Caribbean | AJ970970 | [40] |
| South America | Brazil | AJ970974 | [40] |
| South America | Brazil | AJ970965 | [40] |
| South America | Brazil | AJ970961 | [40] |
| South America | Brazil | JX456414 | [68] |
| South America | Bolivia | JQ926676 | [41] |
| South America | Bolivia | JQ926679 | [41] |
| South America | Bolivia | JQ926683 | [41] |
| South America | Bolivia | JQ926681 | [41] |
| South America | Bolivia | JQ926678 | [41] |
| South America | Bolivia | JQ926677 | [41] |
| South America | Bolivia | JQ926682 | [41] |
| South America | Guyana | HQ688297 | [59] |
| South America | Colombia | KP281728 | Unpublished |
| South America | Colombia | KP281732 | Unpublished |
| South America | Colombia | KM452751 | Unpublished |
| South America | Colombia | KM203228 | [21] |
| South America | Colombia | KM203175 | [21] |
| South America | Colombia | KM203227 | [21] |
| South America | Colombia | KM203244 | [21] |
| South America | Colombia | KM203150 | [21] |
| South America | Colombia | KM203153 | [21] |
| South America | Colombia | KM203143 | [21] |
| South America | Colombia | KM203197 | [21] |
| South America | Colombia | KM203154 | [21] |
| South America | Colombia | KM203179 | [21] |
| South America | Colombia | KM203204 | [21] |
| South America | Colombia | KM203184 | [21] |
| South America | Colombia | KM203212 | [21] |
| South America | Colombia | KM203226 | [21] |
| South America | Colombia | KM203248 | [21] |
